# Combination of basal insulin and GLP-1 receptor agonist: is this the end of basal insulin alone in the treatment of type 2 diabetes?

**DOI:** 10.1186/s13098-018-0327-4

**Published:** 2018-04-03

**Authors:** Rodrigo Oliveira Moreira, Roberta Cobas, Raquel C. Lopes Assis Coelho

**Affiliations:** 1 Instituto Estadual de Diabetes e Endocrinologia, Rua Moncorvo Filho 90, CEP 22280-110 Rio de Janeiro, Brazil; 2 Universidade do Estado do Rio de Janeiro, Rio de Janeiro, Brazil; 3 Novo Nordisk , São Paulo, Brazil

**Keywords:** Type 2 diabetes mellitus, Basal insulin, Glucagon-like peptide 1 receptor agonist, Fixed combination

## Abstract

Glycemic control has been considered a major therapeutic goal within the scope of diabetes management, as supported by robust observational and experimental evidence. However, the coexistence of micro and macrovascular disease is associated with the highest cardiovascular risks which highlights the importance that pharmacological treatment of type 2 diabetes mellitus provides not only glycemic control, but also cardiovascular safety. Basal insulin is a highly effective treatment in reducing fasting blood glucose, but it is associated with considerable risk of hypoglycemia and weight gain. Glucagon like peptide 1 receptor agonists (GLP-1 RAs) are also effective in terms of glycemic control and associated with weight loss and low risk of hypoglycemia. The potential benefits of combining GLP-1RAs with basal insulin are contemplated in the current position statement of several different position statement and guidelines. This article reviews the efficacy and safety of different strategies to initiate and intensify basal insulin, with focus on new fixed ratio combinations of basal insulin with GLP-1 RAs available for use in a single injection pen (insulin degludec/liraglutide and insulin glargine/lixisenatide).

## Background

Glycemic control has been considered a major therapeutic goal within the scope of diabetes management, as supported by robust observational and experimental evidence. Different guidelines [1, 2] have shown the importance of reaching and keeping haemoglobin A1c (A1c) in target; specially to prevent microvascular complications. As diabetes is a progressive disease, intensification of treatment is often needed to achieve and maintain glycemic control [3].

Macrovascular disease is also a major concern in type 2 diabetes mellitus (T2DM) treatment. The coexistence of micro and macrovascular disease was associated with the highest cardiovascular risks [4]. In this sense, it is important that pharmacological treatment of T2DM provides not only glycemic control, but also cardiovascular safety and clinically sound benefits in terms of reducing cardiovascular events.

Basal insulin is a highly effective treatment in reducing fasting blood glucose. However, it is associated with considerable risk of hypoglycemia and weight gain [5]. Because of these clinical concerns, basal insulin initiation and intensification is often delayed by prescribers. On the other hand, glucagon like peptide 1 receptor agonists (GLP-1 RAs) are also effective in terms of glycemic control (both fasting and postprandial) and associated with weight loss and low risk of hypoglycemia. However, their use may cause gastrointestinal side effects, especially nausea, which is dose-related and may be prevented by slow dose escalation [6].

The potential benefits of combining GLP-1RAs with basal insulin are contemplated in the current position statement of both the American Diabetes Association (ADA) [1] and the American Association of Clinical Endocrinologists (AACE) [2]. Although this is an effective strategy, it requires multiple injections. This is why fixed-ratio combination products that require only one injection per day have been developed.

This article reviews the efficacy and safety of different strategies to initiate and intensify basal insulin, with focus on new fixed ratio combinations of basal insulin with GLP-1 RAs available for use in a single injection pen (insulin degludec/liraglutide and insulin glargine/lixisenatide).

## Materials and methods

This article is based on the review of previously published data and does not involve any new studies on human or animal subjects performed by any of the authors.

A PubMed search was conducted using a structured search strategy comprising the following terms: combination GLP-1RA and basal insulin; fixed ratio combination; IDegLira; IGlarLixi. The search also included abstracts published in the last American Diabetes Association (ADA) Scientific Sessions. Current guidelines for T2DM management from ADA and The American Association of Clinical Endocrinologists (AACE) were consulted. We considered for this review articles published in English from 2000 to 2018. The authors’ consensus identified relevant articles. Reference lists of included articles were also screened to identify further relevant studies.

## Results

### Importance of glycemic control and adherence to treatment in T2DM

Considering the increasing expected prevalence of T2DM worldwide [7], strategies to improve metabolic control must be emphasized to reduce diabetes morbidity and its social and economic impact. Early tight glycemic control in T2DM is a well-established recommendation with supporting evidence [3, 4]. Nevertheless, a retrospective analysis of T2DM treatment in UK showed that the mean time to add a second oral agent in patients with A1c above 7.0, 7.5 and 8.0%, was 2.9, 1.9 and 1.6 years, respectively. The mean time to intensification of treatment with insulin was 7.1, 6.1 and 6.0 years for those taking one, two or three oral drugs. Also, the mean A1c level at intensification with either an oral agent or insulin was 8.7, 9.1 and 9.7% for patients taking one, two or three oral agents [8]. These data reinforce the importance of avoiding clinical inertia and identifying the limiting factors that may influence this delay in treatment intensification, particularly to start insulin.

Adherence to treatment in chronic diseases is an old but still relevant problem involving factors related to the treatment regimen, the patient and/or the health care providers. Diabetes is a complex disease that requires not only adherence to medications (including insulin regimens and oral drugs) but also to diet, physical exercise and home glucose monitoring, among others. For more than a decade the World Health Organization (WHO) has published a report highlighting the relevance of considering low adherence to treatment in health policy decisions and its marked economic and health consequences [9]. Studies assessing adherence behaviours in diabetes and its determinants concluded that high adherence was associated with better glycemic control [10, 11] and fewer need of hospital admissions and department visits and also lower overall costs [11]. A systematic review of 76 studies using electronic monitoring to compare mean compliance with different dosing schedule regimens demonstrated an inverse relationship between number of doses per day and compliance [12].

Regarding specificities of diabetes and its treatment, the psychological resistance to insulin regimens is an important issue. This has been investigated in 708 T2DM patients, mean age 57 years-old, 66% women, with a mean duration of diabetes of 7 years attending a diabetes conference in several cities in USA. Interestingly, only 28.2% of the patients were classified as unwilling to initiate insulin. The most common reasons for resistance to initiate insulin were restrictiveness (‘insulin therapy would restrict my life’), low self-efficacy (‘I am not confident I could handle the demands of insulin therapy’), personal failure (‘insulin therapy would mean I had failed’) and permanence (‘once you start insulin you can never quit’) [13]. It is worth mentioning that most of the reasons could be demystified with proper diabetes education and with the new, easier-to-use and painless devices to deliver insulin and monitor blood glucose. In fact, a systematic review aiming to identify factors associated with adherence to insulin therapy in type 1 and type 2 diabetes showed that adherence is generally poor and suggested that more flexible regimen may improve it as well as facilitating insulin deliver by switching to a pen device [14].

### Basal insulin initiation and intensification: current position statements

Regardless of the type of treatment to be instituted, emphasis should be placed on the importance of early glycemic control and avoiding therapeutic inertia. According to the ADA 2018 Guidelines [1], treatment for T2DM should start with monotherapy unless A1c is greater or equal than 9.0%, when dual therapy must be considered. For patients with symptoms, A1c greater or equal than 10% or blood glucose greater or equal than 300 mg/dL, combined injectable therapy should be considered, including basal insulin plus bolus insulin or GLP-1 RA or premixed insulin.

Basal insulin therapy should be considered when introducing dual or triple therapy and should be intensified adding a mealtime insulin or a GLP-1 RA when glycemic control needs to be optimized [1]. In this sense, the combination of basal insulin and GLP-1 RA could already be indicated as first step (in states of very poor glycemic control) or as second step (from monotherapy to triple therapy).

### Basal insulin intensification: mealtime bolus insulin or GLP-1RA?

Until recently, when basal insulin alone was not enough to maintain glycemic control, the main effective intensification option was to add mealtime bolus insulin in the main meal. The rationale is addressing postprandial glucose (PPG) control. PPG control should be addressed as an important target because postprandial hyperglycemia contributes to A1c, especially at lower values, and is considered an independent risk factor for micro and macrovascular complications [15]. However, there are some perceived barriers to insulin therapy intensification. The most important are risk of hypoglycemia, weight gain and a more complex regimen of treatment, with more injections and monitoring procedures every day.

In turn, a GLP-1RA will optimize the prandial endogenous insulin response to control PPG. Thus, it reduces the insulin dose requirement, and also attenuates the weight gain associated with insulin therapy. GLP-1RAs glucose-lowering action is glucose dependent, thus these class is associated with very low risk of hypoglycemia. In turn, nausea is the main adverse event associated with GLP-1RAs class. Slow titration allows a gradual increase in the GLP-1RA dose, thereby helping to avoid nausea.

**Fig. 1:**
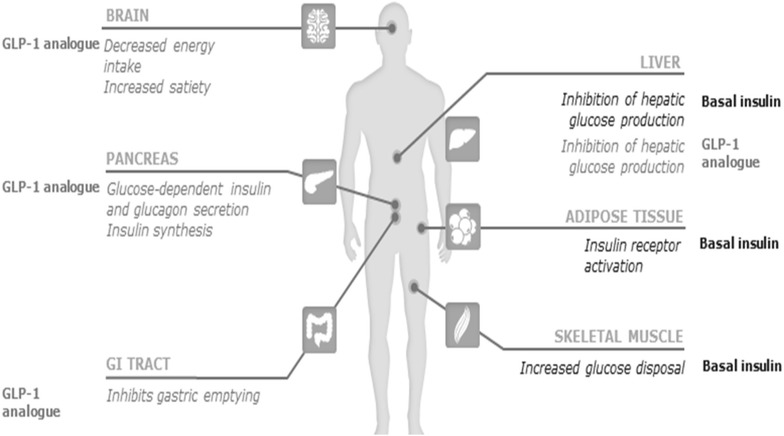
Complementary actions of basal insulin and GLP-1 analogue target the underlying pathophysiology of T2DM (Adapted from references 5 and 6)

As explained, there is a rationale for the combined use of basal insulin and GLP-1RAs. The association can provide the benefit of two highly effective drugs in glycemic control and attenuate adverse events, such as hypoglycemia and weight gain (these benefits will be discussed in detail further in the article). Because of their complementary modes of action, basal insulin and GLP-1RAs together act in most of the defects seen in T2DM, in accordance with its complex physiopathology (Fig. 1).

### Basal insulin and GLP-1RAs: fixed ratio combination

Two fixed combinations of Insulin and GLP1-RA are currently available: IDegLira and IGlarLixi. IDegLira is a fixed-ratio combination of insulin degludec and liraglutide in a 3-mL prefilled injection pen—100 units/mL (U/mL) of degludec and 3.6 mg/mL of liraglutide. Degludec is a long-acting basal insulin (T1/2 = 25 h) which mechanism of protraction is multihexamer formation in the subcutaneous injection depot. Degludec has a predictably flat and stable glucose-lowering action [16]. Liraglutide is a once-daily analogue of human GLP-1 with 97% amino acid sequence homology to endogenous GLP-1. Liraglutide acts on both FPG and PPG excursions; with a half-life of approximately 13 h, which means once-daily dosing [17].

IGlarLixi is a fixed-ratio combination of insulin glargine and lixisenatide, containing 100 U/mL of glargine and 33 lg/mL of lixisenatide in a 3-mL prefilled injection pen. Glargine U100 is a basal insulin with post-injection precipitation that achieves a protracted action (T1/2 = 12.5 h) [5]. Lixisenatide is an exendin-derived GLP-1RA for which PPG lowering is brought about mostly through delayed gastric emptying and reduced glucagon release. Lixisenatide has a half-life of only 2–4 h. This relatively shorter half-life means that PPG control occurs mainly in the meal after injection [18].

### Clinical efficacy

The clinical efficacy and safety of these combination products has been established through a series of phase 3 trials (DUAL clinical program for IDegLira and LixiLan for IGlarLixi). The studies included different populations of patients with DM, including both patients naïve of insulin as well as patients already receiving insulin. The main studies of both clinical programs are detailed below.

#### Clinical trials with IDegLira

##### Insulin naive patients

In the DUAL-1 trial [19], 1663 insulin-naive patients with T2DM in use of metformin and/or pioglitazone were randomized to receive IDeglira, insulin degludec or liraglutide for 26 weeks. The primary endpoint was to prove non-inferiority of the combination compared to degludec and superiority compared to liraglutide. All patients randomized to IDegLira started with 10 U daily. Non-inferiority was proven for IDegLira compared to degludec (ΔA1c − 0.47%; CI − 0.58 to − 0.36%; p < 0.0001) and superiority was proven for IdegLira compared to liraglutide alone (ΔA1c − 0.64%; CI − 0.75 to − 0.53%; p < 0.0001). It is important to notice that there was no limit for degludec dose in this study. The mean dose achieved during titration of IDeglira combination was 38 Units (equivalent of 1.4 mg of liraglutide and 38 IU of degludec) compared to 1.8 mg of liraglutide alone and 53 IU of degludec alone. The IDegLira group used a 28% smaller insulin dose than the degludec group. Also, patients randomized to liraglutide (7%) and to IDegLira (32%) presented significant lower rates of confirmed hypoglycemia than patients randomized to degludec (39%; p < 0.001 vs liraglutida and p = 0.023 vs IDegLira). The main results are summarized in Table 1.

**Table 1:** Studies evaluating the efficacy of IDegLira and IGlarLixi in patients with diabetes mellitus type 2 inadequately controlled with oral medication and insulin naive

Study	DUAL-1			LixiLan-O		
	IDegLira	Degludec	Liraglutide	IGlarLixi	Glargine U100	Lixisenatide
Duration	26 weeks			30 weeks		
Population	1663 T2DM adults, A1c 8.3 ± 0.9; BMI 31.2 ± 4.8 kg/m^2^, metformin ± pioglitazone			1170 T2DM adults, A1c 8.2 ± 0.7; BMI 31.7 ± 4.4 kg/m^2^; metformin ± pioglitazone		
Mean insulin dose (final)	38 ± 13	53 ± 28		39 ± 14	40 ± 14	
ΔA1c	− 1.9 ± 1.1	− 1.4 ± 1.0	− 1.3 ± 1.1	− 1.6 ± 0.1	− 1.3 ± 0.1	− 0.8 ± 0.1
Final A1c (week 30)	6.4 ± 1.0	6.9 ± 1.1	7.0 ± 1.2	6.5 ± 0.8	6.8 ± 0.8	7.3 ± 0.9
Δ body weight (kg)	− 0.5 ± 3.5	+ 1.6 ± 4.0	− 3.0 ± 3.5	− 0.3 ± 0.2	+ 1.1 ± 0.2	− 2.3 ± 0.3
% A1c < 7%	81	65	60	74	59	33
%A1c < 7% without weight gain	46	21	54	43	25	28
%A1c < 7% without hypoglycemia	60	41	58	53	44	30
%A1c < 7% without weight gain or hypoglycemia	36	14	52	32	19	26
Hypoglycemia (%)^a^	32	39	7	26	24	6

^a^Different definitions for hypoglycaemia were used in the two studies. For the DUAL I trial, confirmed hypoglycaemia was defined as the occurrence of episodes requiring assistance (severe), or episodes in which plasma glucose concentration (determined from self-monitored blood glucose) was less than 56 mg/dL, irrespective of symptoms. For the LixiLan-O trial, documented symptomatic hypoglycemia was defined as typical symptoms of hypoglycaemia accompanied by a measured plasma glucose concentration of #70 mg/dL

Patients of DUAL-1 were followed-up for a 26-week extension period [20]. Overall, at week 52, 56.5% of the patients reached the dose of 50 dose-steps (50 IU of insulin degludec plus 1.8 mg of liraglutide) compared to 44% at week 26 in DUAL-1. The mean insulin dose was 37% lower in the IDeglira compared to degludec arm.

A subgroup of the DUAL-1 population (n = 260) was evaluated for parameters of glycemic control measured by CGMS over 72 h and hormonal and beta cell function during a standardized meal test (‘Ensure Plus’—675 kcal, 15% of protein, 57% of carbohydrates, 28% of lipids) [21]. At week 26, the mean dose of liraglutide and insulin degludec was, respectively, 1.5 and 1.8 mg in the IDegLira and liraglutide arms and 43 and 60 IU in the IDegLira and degludec arms. There was a reduction in the increment of post-prandial glycemia (AUC 0–4 h) during the meal test from baseline to week 26 of 21.6% in the IDegLira compared to 4% in the degludec arm (p = 0.0023) and 18.4% in the liraglutide arm (p = 0.7). The insulin secretion ratio (p = 0.048) and static index (p = 0.006) were greater in breakfast and supper for IDegLira compared to degludec but similar to liraglutide (p = 0.45 and 0.895, respectively).

Three other studies were performed with IDegLira in insulin-naïve patients. The DUAL-III study evaluated the efficacy of IDegLira in patients receiving GLP-1 RA and the DUAL-IV study in patients receiving sulphonylurea alone or combined to insulin. The DUAL VI study was designed to evaluate different titration strategies. Different from the DUAL-I study, there are no studies with IGlarLixi in these populations.

In the DUAL-III trial [22], 438 patients with T2DM receiving GLP-1 RA were randomized to receive IDegLira ou to continue with GLP-1 RA therapy. The primary objective was to prove superiority of IDegLira in comparison to continuing with GLP-1 RA therapy. All patients randomized to IDegLira started with 16 dose-steps daily (16 UI of degludec + 0.6 mg of liraglutide). After 26 weeks of treatment, superiority of IDegLira was demonstrated. Patients randomized to IDegLira presented a significant reduction of A1c in comparison to placebo (ΔA1c − 0.94%; p < 0.0001). As expected, the mean chance in weight was + 2.0 kg with IDegLira versus − 0.8 kg with placebo. Also, patients randomized to IDegLira experienced significantly higher rates of hypoglycaemia and nocturnal hypoglycaemia.

In the DUAL IV trial [23], 435 insulin-naïve patients uncontrolled on sulphonylureas with or without metformin were randomized to IDegLira or Placebo. As in DUAL-I study, patients randomized to IDegLira started with 10 dose-steps daily (10 UI of degludec + 0.36 mg of liraglutide). After 26 weeks, patients randomized to IDegLira presented a significant improvement in glucose control in comparison to placebo (mean A1c change: − 0.46% for placebo and − 1.45% for IDegLira (estimated treatment difference = 1.02%) − 1.02% [CI − 1.18 to − 0.87]; p < 0.001). Interestingly, at the end of the trial, the mean dose of IDegLira was 28 dose-steps (28 UI of degludec + 1.0 mg of liraglutide). There was an increase in body weight in patients randomized to IDegLira (+ 0.5 kg) in comparison to a reduction in the placebo group (1.0 kg). As expected, confirmed hypoglycaemia occurred more frequently in IDegLira group (41.7% vs 17.1% in the placebo group).

The DUAL VI trial [24] was designed to compare the safety and efficacy of 02 titration strategies of IDegLira for uncontrolled T2DM patients receiving either metformin or metformin + pioglitazone. Patients were then randomized to once-weekly titration group (based on the mean of 2 fasting SMPG values measured pre-breakfast in the morning of 2 consecutive days) or the twice-weekly titration group (every 3–4 days). No difference was found in A1c reduction, weigh chance or hypoglycaemia rates between the two groups.

##### Insulin treated patients

The DUAL-2 trial [25] was a 26-week, double-blind phase 3 trial, designed to confirm the superiority of IDegLira compared to insulin degludec alone. T2DM adults treated with basal insulin (20–40 IU) and metformin ± sulphonylureas or glinides with A1c between 7.5 and 10% and BMI ≥ 27 kg/m^2^ were included. After randomization, sulphonylureas and glinides were stopped and patients were randomized to receive IDegLira plus metformin or degludec plus metformin. Doses were titrated until a fasting glycemia between 72–90 mg/dL was achieved. As in DUAL-1 study, there was no upper limit for degludec dose. The initial dose of IDegLira was 16 IU (equivalent of liraglutide 0.6 mg and degludec 16 UI). Maximum dose was 50 units of degludec or 50 dose steps IDegLira (50 units degludec plus 1.8 mg liraglutide). Summarized results are presented in Table 2.

**Table 2:** Studies evaluating the efficacy of IDegLira and IGlarLixi in patients with diabetes mellitus type 2 inadequately controlled with basal insulin

Study	DUAL-2		DUAL-5		LixiLan L	
	IDegLira	Degludec	IDegLira	Glargine U100	IGlarLixi	Glargine U100
Duration	26 weeks		26 weeks		30 weeks	
Population	413 T2DM adults, A1c 8.7 ± 0.7%, basal insulin and metformin ± sulphonylureas or glinides; mean basal insulin dose at baseline 29 ± 8		557 T2DM, A1c 8.4/8.2 ± 0.9%, basal insulin (glargine) and metformin; mean basal insulin dose at baseline 31 ± 10		736 T2DM adults, A1c 8.1 ± 0.7%, basal insulin and metformin ± OAD; mean basal insulin dose at baseline 35 ± 9	
Mean insulin dose (final)	45	45	41	66	47	47
ΔA1c	− 1.9	− 0.9	− 1.8	− 1.1	− 1.1	− 0.6
Final A1c (week 30)	6.9	8.0	6.6	7.1	6.9	7.5
Δ body weight (kg)	− 2.7	0.0	− 1.4	+ 1.8	− 0.7	0.7
% A1c < 7%	60	23	72	47	34	13
A1c < 7% without weight gain (%)	NA	NA	50	20	34	13
A1c < 7% without hypoglycemia (%)	NA	NA	54	29	32	19
A1c < 7% without weight gain or hypoglycemia (%)	40	8	39	12	20	9
Hypoglycemia (%)^a^	24	25	28	49	40	42

^a^Different definitions for hypoglycaemia were used in the two studies. For the DUAL 2 and 5 trials, confirmed hypoglycaemia was defined as the occurrence of episodes requiring assistance (severe), or episodes in which plasma glucose concentration (determined from self-monitored blood glucose) was less than 56 mg/dL, irrespective of symptoms. For the LixiLan-L trial, documented symptomatic hypoglycemia was defined as typical symptoms of hypoglycaemia accompanied by a measured plasma glucose concentration of #70 mg/dL

In the DUAL-V trial [26], the combination IDegLira was compared to insulin glargine up-titration in patients with uncontrolled T2DM. The main objective was to prove non-inferiority of IDegLira on A1c levels. The studied population included 557 patients treated with insulin glargine (20–50 IU) and metformin (≥ 1500 mg/day), with A1c levels between 7 and 10% and BMI ≤ 40 kg/m^2^. This was a phase 3 multicenter open-label, treat-to-target trial of 26 weeks of duration. The 278 patients randomized to receive IDegLira stopped glargine use and initiated IDegLira with an initial dose of 16 IU, increasing steps of titration according to fasting glycemia levels. The maximum dose of IDegLira was 50 IU. Patients in the glargine group (n = 279) maintained the insulin glargine once daily and the dose was also titrated (without any upper limit). The titration was performed to achieve a fasting glycemia between 72 and 90 mg/dL (mean of 3 consecutive days). The primary outcome was change in A1c from baseline to week 26 (ΔA1c). The results are summarized in Table 2.

In the DUAL VII trial [27], patients with uncontrolled T2DM receiving insulin were randomized to IDegLira or basal Insulin + Insulin Aspart (basal–bolus therapy). As in DUAL II and V trials, IDegLira was started with 16 dose-steps daily (16 UI of degludec + 0.6 mg of liraglutide). The main finding of the study was that no difference was observed in A1c reduction in IDegLira in comparison to basal–bolus group. On the other hand, significant differences were observed regarding weight chance and hypoglycaemia rates. During treatment period (26 weeks), significant more patients randomized to basal–bolus experienced a symptomatic hypoglycemic event (52.6%) in comparison to IDegLira (19.8%). In general, there was a 89% reduction in severe or confirmed hypoglycaemia compared to basal–bolus. Mean basal insulin dose with basal–bolus increased from 34 units in week 1–52 units at 26 weeks compared to a mean dose of 40 units with IDegLira (corresponding to 40 units degludec/1.44 mg liraglutide). Finally, the mean body weight decreased by 0.9 kg with IDegLira and increased with basal–bolus by 2.6 kg.

##### Additional analysis with IDegLira

A post hoc analysis [28] was performed to investigate if IDegLira was consistently effective in T2DM patients independently of their baseline A1c levels and duration of diabetes. This analysis included data from DUAL-1 extension (52 weeks) and DUAL-2 (26 weeks). Four categories of A1c were created: ≤ 7.5%; 7.5 to ≤ 8.5%; 8.5 to ≤ 9 and > 9%. Across all categories, A1c reductions were significantly greater with IDeglira compared with the degludec or liraglutide alone in DUAL-1.

Reductions in A1c levels were similar in any duration of diabetes in DUAL-1 and 2. In DUAL-1 the A1c reduction from baseline with IDegLira was somewhat higher (estimated difference of 0.21%) in patients in use of pioglitazone and metformin compared to those with metformin alone (ΔA1c for IDegLira − 1.8% in the metformin subgroup and − 2.1% in the metformin plus pioglitazone subgroup; p = 0.002). For degludec and liraglutide alone groups, the differences of the A1c reductions were not significant between baseline oral drugs subgroups. In DUAL-2, the A1c reductions were significantly different across the subgroups of baseline treatment. However, no difference was observed according to pre-study insulin dose.

IDegLira was also indirectly compared to alternative strategies of glycemic control intensification in patients with T2DM treated with basal insulin [29]. This was an analysis of 5 trials: 199 patients with IDegLira, 225 patients with basal insulin plus liraglutide, 56 patients with basal–bolus insulin, 329 patients with glargine. Results are presented in Table 2. The mean differences in A1c and body weight between IDegLira and basal–bolus therapy and IDeglira and basal glargine were − 0.3% and − 6.89 kg and − 0.65% and − 4.04 kg respectively. The OR of achieving an A1c < 7% was 2.06 for IDegLira compared to basal insulin plus liraglutide and 3.91 for IDegLira compared to basal glargine. The OR of achieving an A1c < 7% without hypoglycemia was 16.05 for IDegLira versus basal–bolus therapy and 4.53 for IDegLira versus basal glargine but there was no difference between IDeglira compared to basal insulin plus liraglutide.

#### Clinical trials with IGlarLixi

##### Insulin naive patients

In the LixiLan-O trial [30], 1170 insulin-naive patients with T2DM in use of metformin and/or pioglitazone were randomized to receive once daily insulin glargine, lixisenatide or IGlarLixi for 30 weeks. Both groups titrated to fasting plasma glucose < 100 mg/dL up to a maximum insulin dose of 60 units/day, or to once-daily lixisenatide (20 mg/day) while continuing with metformin. The primary endpoint was A1c change. As for the DUAL-1 study, the primary endpoint was to prove non-inferiority of the combination compared to glargine and superiority compared to lixisenatide. Superiority was proven for IGlarLixi compared to lixisenatide (ΔA1c − 0.8%; CI − 0.9 to − 0.7%; p < 0.0001) and to glargine alone (ΔA1c − 0.3%; CI − 0.4 to − 0.2%; p < 0.0001). It is important to notice that the maximum glargine once daily dose was capped at 60 IU. The mean final basal insulin daily dose was similar between both groups. Interestingly, the incidence of symptomatic documented hypoglycemia was similar with IGlarLixi and glargine (26 and 24%, respectively) and lower in the lixisenatide group (6%). The main results are summarized in Table 1.

##### Insulin treat patients

The LixiLan-L trial [31] was a 30-week, double-blind trial, designed to confirm the superiority of IGlarLixi in comparison to glargine alone in patients with T2DM inadequately treated with a stable dose of insulin glargine and any other OAD. The mean starting A1c was 8.5%. Eligible patients entered run-in phase where any OAD other than metformin were stopped and patients were prescribed glargine. After 6 weeks, patients were then randomized to IGlarLixi or glargine. Doses were titrated until a fasting glycemia between 80–100 mg/dL was achieved. As in LixiLan-O trial, the maximum glargine dose was capped at 60 IU. The mean results are summarized in Table 2.

### Cardiovascular safety

In terms of cardiovascular safety, IDegLira contains liraglutide, the only Federal Drug Administration (FDA) approved GLP-1RA shown to reduce cardiovascular risk. Regarding the incidence rates of major adverse cardiovascular events (MACE), liraglutide was superior to placebo in the LEADER cardiovascular outcomes trial (CVOT). The primary outcome (the first occurrence of death from cardiovascular causes, nonfatal myocardial infarction, or nonfatal stroke) was less frequent in the liraglutide group (13.0% vs. 14.9%, p < 0.001 for noninferiority; p = 0.01 for superiority). Liraglutide-treated patients had also a lower risk of death from cardiovascular causes and from any cause [32]. In the ELIXA CVOT, lixisenatide was found to be non-inferior to standard of care plus placebo in terms of risk of the composite endpoint (cardiovascular death, myocardial infarction, stroke, or hospitalization for unstable angina)—13.4% in the lixisenatide group and 13.2% in the placebo group (hazard ratio, 1.02; 95% confidence interval [CI], 0.89–1.17) [33].

Cardiovascular safety also has been established for both insulin degludec and glargine. The DEVOTE study showed that degludec was non-inferior to glargine U100 with regard to incidence rates of MACE [34]. Cardiovascular safety of glargine U100 has been previously shown in ORIGIN study [35].

### Clinical use and practical aspects

Patients who need further intensification of therapy but have had it delayed because of concerns about potential weight gain and hypoglycemic episodes would probably benefit from fixed-ratio combination of basal insulin and GLP-1Ras [19, 22, 23, 25–27, 30, 31]. Also, adherence and treatment satisfaction may increase in a regimen that does not increase the daily number of injections, while still promoting weight neutrality or loss and reduced frequencies of hypoglycemic episodes. In addition, patients will benefit from the relative simplicity of these products. A patient who uses a basal–bolus insulin regimen could potentially reduce their weekly number of injections from 28 to 7 by switching to a fixed ratio combination basal insulin/GLP-1RA option. To health care providers, the combination is potentially helpful to avoid clinical inertia and address adherence issues on insulin intensification.

IDegLira is administered once-daily through a subcutaneous injection, given at the same time of the day without the need to coincide with mealtime. IDegLira initial dosing is 16 U for patients previously in use of basal insulin, delivering 16 U of degludec and 0.58 mg of liraglutide. The same starting dose is also recommended for patient already taking a GLP1-RA. For insulin naïve, starting dose is 10 U; with a maximum dose of 50 U (50 U of degludec and 1.8 mg of liraglutide). Prebreakfast self-monitoring plasma glucose (SMPG) results and individual patient’s glycemic target range should be used to titrate the dose [36].

IGlarLixi is also administered as a once-daily subcutaneous injection within 1 h of the first meal of the day. The recommended initial dosing of IGlarLixi for patients previously uncontrolled on lixisenatide or on less than 30 IU basal insulin is 15 U (15 U of glargine and 5 mcg of lixisenatide). For patients previously uncontrolled with 30–60 IU of basal insulin, the recommended starting dose of IGlarLixi is 30 IU (30 U of glargine and l0 mcg of lixisenatide). The maximum dose is 60 IU glargine/20 mcg lixisenatide. In a similar way as other insulin containing products, the dose should be titrated once a week according to the individual patient’s glycemic target range [37].

## Conclusion

T2DM is a progressive disease and treatment intensification is often required to achieve and maintain glycemic control [1, 2]. Basal insulin and GLP-1RAs address several of the defects seen in T2DM physiopathology. When insulin/GLP-1RA fixed-ratio combinations are compared with basal insulin, a superiority in reducing HbA1c is observed, with overall benefit also in weight neutrality or weight loss, reduced hypoglycemia risk, and reduced insulin-dose requirement [19, 25–27, 30, 31]. Also, fixed-ratio combination has the advantage of a less complex treatment regimen, with only one injection a day. Cardiovascular safety in terms of MACE risk reduction, which is always a concern in T2DM, is well established for the monocomponents [32–35]. Data from robust clinical trials highlight the great potential of these products. In summary, fixed ratio combination of GLP-1RA and basal insulin are potentially helpful tools for the treatment of patients with T2DM as a result of its favorable safety and efficacy profile, particularly in patients who are overweight and uncontrolled on OADs or basal insulin. For these patients, basal insulin alone may not be the preferred option.
